# Neonatal subpial hemorrhage: Padua experience and systematic review

**DOI:** 10.1007/s00431-025-06021-y

**Published:** 2025-03-24

**Authors:** Cristina Impieri, Claudio Ancona, Benedetta Bortolatto, Irene Laghetto, Sofia Galzignato, Margherita Nosadini, Irene Toldo, Ignazio D’Errico, Stefano Sartori, Giulia Calignano, Maria Elena Cavicchiolo, Elena Cavaliere

**Affiliations:** 1 Department of Women’s and Children’s Health, University of Padua, Padua, Italy; 2 Paediatric Neurology and Neurophysiology Unit, Department of Women’s and Children’s Health, University Hospital of Padua, Padua, Italy; 3 Neuroradiology Unit, University Hospital of Padua, Padua, Italy; 4 Department of Developmental Psychology and Socialization (DPSS), University of Padua, Padua, Italy; 5 Neonatal Intensive Care Unit, Department of Woman’s and Child’s Health, University Hospital of Padua, Padua, Italy

**Keywords:** Subpial hemorrhage, Leptomeningeal hemorrhage, Hemorrhagic stroke, Newborn, Neonate

## Abstract

Subpial hemorrhage (SPH) is a rare subtype of intracranial hemorrhage, predominantly affecting term neonates and often associated with cortical-subcortical infarction. We described the epidemiology of SPH by analyzing cases referred to our hospital and concurrently conducting a systematic review of the cases reported in the literature. We also illustrated factors associated with adverse outcomes. A retrospective study was conducted on neonates with SPH referred to our hospital from 2013 to 2023 (cohort 1). Additionally, a systematic literature review on neonatal SPH was performed using PubMed, Scopus, Cochrane, and Web of Science up to April 2024 (cohort 2). Cohorts 1 and 2 were pooled for combined analysis. A total of 173 cases were analyzed, 10 original cases (cohort 1) and 163 literature cases (cohort 2). Ninety-two percent was term/late preterm neonates (59% male). Clinical presentations included seizures (36%), apnea (36%), and encephalopathy (18%). Ninety-four percent was diagnosed with brain magnetic resonance imaging and/or cranial ultrasound. Lesions were located in the temporal lobe in 60%, with infarctions adjacent to SPH in 90%. Sixteen percent died, 53% was diagnosed with neurological impairment, and 8% with epilepsy. In a subcohort of 67 patients (cohort 3) with available individual data (10/10 from cohort 1, 57/163 from cohort 2), low birth weight (LBW), seizures, neonatal infections, and parenchymal hemorrhage were significantly associated with adverse outcomes.

*Conclusion*: Neonatal SPH is rare, predominantly located in the temporal lobe, and frequently presents with seizures and apneas. Neurologic sequelae are common, and parenchymal hemorrhage was strongly associated with neurological impairment in our study.

**Table:** 

**What is Known:** • *Subpial hemorrhage is a rare subtype of intracranial extra-axial bleeding, often associated with cortical-subcortical infarction in the adjacent parenchyma, predominantly affecting male term neonates. The temporal lobe is the most commonly involved area, frequently exhibiting the "yin-yang sign" on brain MRI.*	
**What is New:** • *This is the first systematic review of neonatal subpial hemorrhage, emphasizing a distinctive clinical presentation marked by seizures and apneas (potentially of ictal origin), consistent with a high prevalence of temporal lobe involvement. Prognostically, a significant incidence of neurological impairment was observed, and the occurrence of parenchymal hemorrhage adjacent to subpial hemorrhage was strongly associated with adverse outcomes.*

**What is Known:**

• *Subpial hemorrhage is a rare subtype of intracranial extra-axial bleeding, often associated with cortical-subcortical infarction in the adjacent parenchyma, predominantly affecting male term neonates. The temporal lobe is the most commonly involved area, frequently exhibiting the "yin-yang sign" on brain MRI.*

**What is New:**

• *This is the first systematic review of neonatal subpial hemorrhage, emphasizing a distinctive clinical presentation marked by seizures and apneas (potentially of ictal origin), consistent with a high prevalence of temporal lobe involvement. Prognostically, a significant incidence of neurological impairment was observed, and the occurrence of parenchymal hemorrhage adjacent to subpial hemorrhage was strongly associated with adverse outcomes.*

## Introduction

Subpial hemorrhage (SPH) is a rare subtype of intracranial extra-axial bleeding predominantly affecting term neonates [1]. It is often associated with parenchymal venous infarction with or without a hemorrhagic component [2].

Historically, SPH was difficult to detect and often grouped with subarachnoid hemorrhages under the terms “leptomeningeal hemorrhages,” “superficial lobar hemorrhage,” “pial hemorrhage,” or “extra-axial bleed with underlying infarct” [3].

The first report of SPH was published by Friede in 1972, based on autopsies of nine infants, identifying a unique type of bleeding between the pia mater and cortical tissue [4]. Advances in brain magnetic resonance imaging (MRI) have improved early detection and understanding of SPH. In 2004, Huang and Robertson confirmed SPH in seven neonates via brain MRI [5]. Cain et al. later reported 17 cases, indicating a deep venous, non-arterial pattern of hemorrhagic ischemia [6].

SPH pathophysiology still remains unclear. SPH involves blood accumulation between the pia mater and the cortical surface, due to injury to glia limitans end-feet and rupture of fragile subpial vessels. It is associated with focal cortical-subcortical cytotoxic edema adjacent to SPH due to the congestion or thrombosis of the superficial venous medullary system. Deep medullary veins could also be involved through transcerebral veins and anastomotic medullary veins. It remains unknown whether SPH originates from primary cerebral venous infarction caused by vascular stressors like infections or birth trauma which damage immature superficial medullary veins leading to congestion and thrombosis and secondary bleeding, or whether the initial hemorrhage in subpial space itself provokes compression and obstruction of cortical venous outflow, subsequently causing a venous infarction of the underlying brain parenchyma [7–10].

SPH typically occurs in male term/late preterm neonates [11, 12].

Common risk factors include chorioamnionitis, maternal hypertension/preeclampsia, fetal distress, asphyxia, neonatal sepsis, and coagulation abnormalities [1, 6].

Clinically, SPH often presents with seizures, especially motor onset seizures, and apneas, possibly of ictal origin. The temporal lobe is the most commonly affected area, often showing a characteristic yin-yang sign on brain MRI [1, 3]. Interestingly, intracranial temporal hemorrhage has been described as related to ictal apneas [13]. Death is reported in patients with severe comorbidities such as congenital heart disease (CHD) or renal failure [6].

Despite its significance, SPH remains underrecognized. Further research is needed to understand its pathophysiology and develop treatment protocols. Current knowledge primarily comes from case reports and series, showing significant variability in diagnosis, treatment, and prognosis.

The aim of this study was to describe risk factors, clinical and neuroradiological presentations, treatments, and outcomes of neonates with SPH by analyzing the cases referred to our Hospital and concurrently conducting a systematic review of those reported in the literature. We also illustrated factors associated with adverse outcomes.

## Materials and methods

We conducted a retrospective case series study on neonates with SPH who were referred to the Department of Children and Women’s Health at Padua University Hospital from July 2013 to December 2023 (cohort 1).

A systematic literature review on neonates with SPH was performed on PubMed, Scopus, Cochrane, and Web of Science according to the PRISMA guidelines by three independent researchers (C.I., B.B., I.L.) up to April 2024 (cohort 2).

The search terms were (“leptomeningeal hemorrhage” OR “leptomeningeal haemorrhage” OR “subpial hemorrhage” OR “subpial haemorrhage” OR “haemorrhagic stroke” OR “hemorrhagic stroke”) AND (neonate* OR newborn*).

Only articles written in English were included. We considered papers reporting at least two clinical or demographic data points on patients aged 0–28 days (or up to 44 weeks of gestational age) with a histological or radiological (brain MRI and/or cranial ultrasound) diagnosis of SPH.

To standardize data, all authors of eligible studies were asked to complete an Excel spreadsheet (Microsoft, Redmond, WA, USA) with patients’ data. One author responded to our request. Two independent researchers (C.I. and S.G.) completed the same datasheet for cohort 1.

Data collection focused on demographics, comorbidities, clinical and radiological features, treatment, and outcomes for both cohort 1 and cohort 2. A pediatric neuroradiologist (I.DE.) analyzed all brain MRI scans of cohort 1.

Data collection was subject to data availability; therefore, in the “Results”, denominators may differ.

The study received the local Ethics Committee approval (Protocol no. 1653P), and each parent signed an informed consent for data collection and extraction for the study. The study was registered in the International Prospective Register of Systematic Reviews (PROSPERO) (ID CRD42024597032).

### Statistical analysis

Cohort 1 and cohort 2 were pooled and described together as the study population (SP). Continuous variables were reported using mean and standard deviations or median and interquartile range and categorical variables with absolute number and percentage (relative frequencies).

Within the SP, we identified a subset of patients with available individual patient data (IPD), which included all patients from cohort 1 and those from cohort 2 with IPD (referred to hereafter as subcohort 2). This subset was designated as cohort 3. In cohort 3, we examined the frequency distribution of the following selected variables: gestational age, female gender, vaginal delivery, LBW (less than 2500 g or 5 pounds, 8 oz), absence/presence of asphyxia, birth trauma (resulting from effects of mechanical forces — compression or traction — imparted on the child’s head during birth) seizure, apnea, encephalopathy (a neonatal syndrome with subnormal level of consciousness and depression of tone and reflexes) temporal location, multifocal (two or more brain regions) SPH, parenchymal hemorrhage (venous infarction complicated with an hemorrhagic component), CHD (both cyanotic and acyanotic structural malformation of the heart or of the big vessels near the heart present at birth), sepsis/neonatal infection, death, and neurological impairment: limited or impaired capacity in any of the following areas: cognitive function, sensory and motor skills, language, emotional regulation, social skills, or basic life functions death or neurological impairment, encephalomalacia at the last follow-up.

Pearson’s correlation coefficients were calculated to assess linear relationships between variables. Analysis was conducted using (R Core Team, 2021) [14] and packages such as ggpubr [15] for correlation tests and dplyr [16] for data management. Statistical significance was set at alpha < 0.05. Only statistically significant variables were then considered. Additionally, the same clinical and neuroradiological factors were associated with indicators of adverse outcomes (death, neurological impairment, death or neurological impairment) combining cohort 1 and subcohort 2 using Pearson’s bivariate correlation analysis.

## Results

From July 2013 to December 2023, 10 newborns with SPH were referred to our center (cohort 1).

**Fig. 1:**
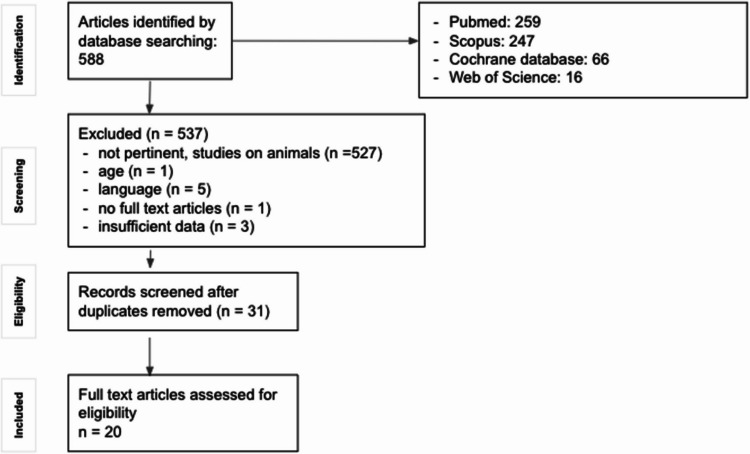
Identification of studies (PRISMA)

Our literature search identified 588 articles, of which 537 were excluded (527 not pertinent/animal studies, 1 different age cohort, 5 foreign languages, 1 no full text, 3 insufficient data). We excluded 31 duplicated and 20 eligible full-text articles reporting on 163 neonates diagnosed with SPH (cohort 2). Combined with 10 newborns of cohort 1, a total of 173 patients (cohort 1 + cohort 2, SP) were analyzed (Fig. 1; Table 1).

**Table 1:** Key information of 20 included studies

Article reference	Study design	Sample size	Data collection year	Where the study took place	Context of the research	Conclusions
Friede et al. [4]	Autopsy neuropathology case series	9, available IPD	1970–1976	Cleveland, Ohio, USA	Neuropathology case series focusing on the characterization of brain hemorrhagic lesions	The first neuropathological description of SPH The authors identified SPH in a significant proportion of infants with perinatal intracranial hemorrhage
Govaert et al. [22]	Autopsy neuropathology case series	1, available IPD	-	Gent, Belgium London, UK	Neuropathology case series of fetal and neonatal intracranial hemorrhages in patients with alloimmune thrombocytopenia	In one patient, the findings were sufficiently specific to identify SPH
Huang et al. [5]	Retrospective case series analysis	7, available IPD	-	Boston, USA	Retrospective case series analysis of neuroimaging and clinical features in healthy-term neonates with spontaneous leptomeningeal hemorrhage	The authors confirmed SPH in seven neonates via brain MRI Hemorrhages were most frequently localized to the temporal lobe and near cranial sutures, often associated with overlying soft-tissue swelling and adjacent areas of restricted diffusion. This pattern suggested that local trauma, resulting in venous compression or occlusion, plays a key role in the pathophysiology of SPH
Slaughter et al. [23]	Retrospective case series analysis	7, available IPD	2000–2007	Cincinnati, Ohio, USA Little Rock, Arkansas, USA	Case series of neonates with unilateral temporal lobe infarct	Temporal lobe hemorrhagic infarcts in neonates, potentially resulting from superficial temporal venous thrombosis, are associated with generally positive outcomes. Nevertheless, extended follow-up into school age is advised to assess the potential for future cognitive or behavioral challenges
Larsen et al. [9]	Retrospective case series analysis	2, available IPD	-	Oxford, UK	Neuropathology case series of 7 infants with subcortical clefts or hemorrhages	Subcortical and subpial hemorrhages may be one indicator of cerebral vein or sinus thrombosis (CVST) and should prompt a search for this etiology
Joel Fluss et al. [35]	Review	1, available IPD	-	Geneva, Switzerland Saint-Étienne, France	Review of perinatal stroke types with 6 case reports, including one featuring SPH in the neonatal period	The term “perinatal stroke” encompasses distinct early brain injuries, characterized by differences in mechanisms, timing, risk factors, and, most importantly, their impact on the developing brain
Tamura et al. [17]	Retrospective case series analysis	2, available IPD	-	IIbaraki, Japan	Case series of surgical treatment in infants with spontaneous parenchymal hemorrhage	The authors suggest that removing a small portion of subdural hemorrhage (SDH) may be both effective and sufficient to alleviate severe symptoms of increased intracranial pressure in term neonates with massive spontaneous parenchymal hemorrhage. 2 cases of SPH were reported
Cain et al. [6]	Retrospective case series analysis	17, available IPD	-	Aurora, Colorado, USA	Monocentric case series of neonatal SPH to enhance understanding of this condition	Fetal distress was observed more often than birth trauma, suggesting that SPH likely occurs in utero rather than as a result of delivery. Venous ischemia may play a significant role in the pathogenesis of SPH
Assis et al. [3]	Retrospective case series analysis	16, pooled data	2006–2020	Calgary, Canada	Cranial ultrasound and MRI characterization, along with associated outcomes, of SPH in neonates	Ultrasound effectively detects SPH and underlying cerebral infarcts identifiable on MRI, except in cases of very small lesions. This condition is also observed in extremely and very preterm infants, who are at a significantly higher risk of severe neurological outcomes
Hausman-Kedem et al. [25]	Retrospective case series analysis	4, available IPD	2016–2020	Tel Aviv, Israel	Investigation of the role of rare genetic variations in unexplained cases of perinatal intracranial hemorrhage	This study suggests a clinically significant diagnostic yield of whole-exome sequencing (WES) in cases of apparently idiopathic perinatal intracerebral hemorrhage, supporting its use in the evaluation of such cases
Karthikeyan et al. [21]	Case report	1, available IPD	-	Pondicherry, India	A case report of a term neonate presenting with seizures, with SPH in the temporal region. This patient was diagnosed with factor XIII deficiency	The diagnosis of SPH should prompt an evaluation for underlying coagulopathies, which may represent the primary cause, as demonstrated in this case
Dabrowski et al. [11]	Retrospective case series analysis	31, pooled data	2009–2020	Baltimore, Maryland, USA	An analysis of risk factors, clinical presentation, hemorrhage size, and long-term outcomes in a cohort of neonates diagnosed with SPH	SPH exhibits distinct imaging and clinical characteristics, suggesting unique pathophysiological mechanisms and implications for long-term outcomes
Anderst et al. [24]	Retrospective case series analysis	1, available IPD	2003–2007	International Paediatric Stroke Study, children with CSVT from 10 countries	To determine the frequency of SDH in children with CSVT, identify factors linked to both CSVT and SDH, and investigate whether observed associations support the hypothesis that CSVT contributes to SDH development	Nearly all 20 subjects with CSVT and SDH had known risk factors for both CSVT and SDH. This study found no evidence to support the hypothesis that CSVT directly causes SDH
Pinto et al. [2]	Retrospective case series analysis	10, pooled data	2010–2020	Porto, Lisboa, Coimbra, Portugal	To characterize a cohort of neonates with SPH and improve the knowledge of this rare type of neonatal stroke	SPH can occur in both late and early preterm neonates, with outcomes varying across cases. Notably, preterm infants do not exhibit worse outcomes compared to term neonates
Matsubara et al. [28]	Retrospective case series analysis	2, available IPD	-	Amagasaki, Japan	Two case reports of term newborns with SPH of the temporal lobe	Both cases exhibited SPH along the medial temporal lobe, suggesting external trauma during delivery as a potential cause. However, other factors, including coagulopathy, may also contribute to SPH pathophysiology
Zhuang et al. [1]	Retrospective case series analysis	34, pooled data	2016–2022	Yuhua District, Changsha, China	A retrospective case series of 34 neonates with SPH, focusing on the imaging features, clinical factors, and outcomes	The authors identified three distinct patterns of SPH, with pattern C, characterized by association with parenchymal hemorrhage, being the most severe
LIm J et al. [36]	Retrospective case series analysis	16, pooled data	2012–2022	Seoul, Korea	This retrospective case series aims to describe the sonographic features of SPH in neonates	SPH can be detected and suspected based on its sonographic features and accompanying findings
Hong et al. [12]	Case report	1, available IPD	-	University of Queensland, Australia	Case report of a term newborn with a temporal lobe SPH	This case report contributes to the existing body of knowledge on SPH
Kattapuram et al. [37]	Case report	1, available IPD	-	Washington, District of Columbia, USA	SPH in an extremely premature infant with ultrasound imaging and MRI follow-up	In this patient SPH was more extensive compared to the predominantly focal and unilateral presentation seen in term and late preterm infants, likely due to the patient’s extreme prematurity
Taori et al. [10]	Case report	1, available IPD	-	Madhya Pradesh, India	Frontal lobe SPH in a term newborn with uneventful pregnancy	In cases of isolated SPH without concurrent brain injury, patients typically show near-complete recovery on follow-up and have an excellent prognosis. Treatment is symptomatic, focusing on appropriate antiepileptic medication

*CSVT* cerebral sinovenous thrombosis, *IPD* individual patient data, *MRI* magnetic resonance Imaging, *SDH* subdural hemorrhage, *SPH* subpial hemorrhage

Cohort 3 included 67 patients (10 patients from cohort 1 and 57 from cohort 2, subcohort 2).

### Description of the SP (cohort 1 + 2, n = 173)

#### Demographics

The majority of the SP was born at term (> 37 gestational weeks, GW) or moderate to late preterm (32–36 + 6 GW) (158/171, 92%), and 85/144 (59%) were male. Most of the deliveries (95/130, 73%) were vaginal, with 11/95 (12%) being assisted births. One hundred nineteen/147 (81%) neonates had a birth weight > 2.5 kg. The majority had a 5-min Apgar score > 7 (143/152, 94%) (Table 2).

**Table 2:** Patient demographics, risk factors, and clinical presentation at onset and treatment

		Cohort 1 *n* 10 *N* (%)	Cohort 2 *n* 163 *N* (%)	Subcohort 2 *n* 57 *N* (%)	Cohort 1 + 2 *n* 173 *N* (%)
Demographics	Extremely preterm (< 28 GW)	0	3/161 (2%)	2/57 (4%)	3/171 (2%)
	Very preterm (28–32 GW)	2/10 (20%)	8/161 (5%)	3/57 (5%)	10/171 (6%)
	Neonates born between 32 and 36 6/7 weeks	2/10 (20%)	33/161 (20%)	5/57 (9%)	35/171 (20%)
	Term (> 37 GW)	6/10 (60%)	117/161 (73%)	45/57 (80%)	123/171 (72%)
	Male	3/10 (30%)	82/134 (61%)	18/27 (66%)	85/144 (59%)
	Birth weight > 2500 g	6/9 (67%)	113/138 (82%)	33/41 (80%)	119/147 (81%)
	Vaginal delivery	6/10 (60%)	89/120 (74%)	33/44 (75%)	95/130 (73%)
	Assisted birth	0	11/89 (12%)	0	11/95 (12%)
	5′ Apgar > 7	7/9 (78%)	136/143 (95%)	29/38 (76%)	143/152 (94%)
	1′ Apgar score (mean; median; range)	7.22; 8; 3–10	6.10; 7,25; 0–9	6.14; 7; 0–9	6.3; 7.5; 0–10
	5′ Apgar score (mean; median; range)	8.25; 8,5; 5–10	7.68; 9; 1–10	7.74; 9; 1–10	7.80; 9; 1–10
	10′ Apgar score (mean; median; range)	8.85; 8.5; 7–10	8.97; 9.5; 4–9.5	NA	8.93; 9.5; 4–10
Risk factors					
*Maternal/pregnancy*	Maternal age in years (mean; median; range)	30.75; 31; 27–34	30.93; 31; 17–44	30.94; 31, 17–44	30,91; 31; 17–44
	Chorioamnionitis	1/10 (10%)	9/117 (8%)	2/42 (5%)	10/127 (8%)
	PROM	2/10 (20%)	5/117 (4%)	2/42 (5%)	7/127 (6%)
	Oligohydramnios	0	6/118 (5%)	2/43 (5%)	6 /128 (4%)
	Maternal infection/fever	0	5/116 (4%)	5/41 (12%)	5/126 (4%)
	Hyper/preeclampsia/eclampsia/HELLP	1/10 (10%)	12/117 (10%)	6/42 (14%)	13/127 (10%)
	Gestational diabetes	1/10 (10%)	3/100 (3%)	1/32 (3%)	4/110 (4%)
*Perinatal*	Asphyxia	1/10 (10%)	10/84 (12%)	3/45 (7%)	11/94 (12%)
	HIE	1/10 (10%)	20/150 (13%)	2/44 (5%)	21/160 (13%)
	Birth trauma	1/10 (10%)	32/117 (27%)	0	33/127 (26%)
	Neonatal resuscitation	4/10 (40%)	15/64 (23%)	15/49 (31%)	19/74 (26%)
	Intubation	4/10 (40%)	15/40 (38%)	15/41 (37%)	19/50 (38%)
*Neonatal*	Hypoglycemia	4/10 (40%)	1/163 (< 1%)	1/57 (2%)	5/173 (3%)
	CHD	1/10 (10%)	6/150 (4%)	2/43 (5%)	7/160 (4%)
	Neonatal sepsis/infection	2/10 (20%)	18/148 (12%)	3/41 (7%)	20/158 (13%)
Coagulation abnormalities	Acute/transient^a^	6/6	35/135 (26%)	20/34 (59%)	41/141 (29%)
	Chronic/permanent^b^	1/1	7/30 (23%)	7/30 (23%)	8/31 (26%)
Clinical characteristics	Onset of symptoms in days (mean; median; range)	1.34; 1; 1–2.9	2; 1; 1–14	2.03; 1; 1–14	1.9; 1; 1–14
	Asymptomatic	1/10 (10%)	6/163 (4%)	0	7/173 (4%)
	Seizures	6/10 (60%)	57/163 (35%)	20/57 (35%)	63/173 (36%)
	Apnea	5/10 (50%)	57/163 (35%)	29/57 (51%)	62/173 (36%)
	Cardiorespiratory failure	0	15/163 (9%)	1/57 (2%)	15/173 (9%)
	Encephalopathy	1/10 (10%)	30/163 (18%)	7/57 (12%)	31/173 (18%)
	Bradi/tachi	4/10 (40%)	2 /163 (1%)	2/57 (4%)	6/173 (3%)
	Cyanosis	3/10 (30%)	14/163 (9%)	9/57 (16%)	17/173 (10%)
	Hyperthermia	2/10 (20%)	3/163 (2%)	0	5/173 (3%)
	Dyspnea	6/10 (60%)	23/163 (14%)	8/57 (14%)	29/173 (17%)
	Jaundice	0	14/163 (9%)	0	14/173 (8%)
Treatment	Antiseizure medication	8/10 (80%)	16/26 (62%)	9/21 (43%)	24/36 (66%)
	Antibiotics	7/10 (70%)	4/36 (11%)	3/20 (15%)	11/46 (23%)
	Surgery	1/10 (10%)	14/161 (9%)	5/55 (9%)	15/171 (9%)

*CHD* congenital heart disease, *g* grams, *GW* gestational week, *HELLP* hemolysis, elevated liver enzymes and low platelets, *HIE* hypoxic ischemic encephalopathy, *PROM* premature rupture of membranes

^a^Acute and transient coagulation abnormalities refer to an alteration of the coagulopathy panel test due to acquired temporary disorder, often systemic in nature, such as sepsis, infection, or vitamin K deficiency

^b^Chronic coagulation abnormalities refer to a permanent coagulopathy often due to a genetic origin

#### Risk factors

Median maternal age was 31 years (range 17–44); in Table 2, the prevalence of maternal/gestational comorbidities is reported.

Perinatal asphyxia was documented in 11/84 (12%) patients, with all but 3 subsequently diagnosed with hypoxic-ischemic encephalopathy (HIE). Birth trauma occurred in 33/127 (26%) newborns. Neonatal resuscitation and intubation were performed in 19/74 (26%) and 19/50 (38%) neonates, respectively. Comorbidities included hypoglycemia (5/173, 3%), infection/sepsis (20/158, 13%), and CHD (7/160, 4%).

Acute or transient coagulation abnormalities were found in 41/141 (29%) neonates, while chronic abnormalities were present in 8/31 (26%). Abnormal coagulopathy panel test was identified in 5/28 infants: one with antithrombin 3 deficiency, one with factor VII deficiency, one with factor XIII deficiency, one with mutation on methylenetetrahydrofolate reductase and plasminogen activator inhibitor 1, and one with a pathogenic variant of Von Willebrand factor and of factor 11.

In cohort 1, three patients underwent a multigene panel for “cerebral microangiopathy” (still in progress) and 1 an array-CGH (still in progress) and FMR1 gene mutation analysis (negative).

#### Clinical characteristics

Symptom onset was between 1 and 14 days of life (median 1 day). The most prevalent clinical symptoms/signs were apneic events (62/173, 36%), seizures (63/173, 36%), encephalopathy (31/173, 18%), and dyspnea (29/173, 17%). Seven out 173 patients were asymptomatic: one of cohort 1 was retrospectively diagnosed at the onset of post-stroke epilepsy at 2 years of age; the others were identified incidentally via cranial ultrasound followed by a brain MRI confirmation.

In cohort 3, 14/67 (21%) presented with seizures, 13/67 (19%) with seizures and apneic events, and 21/67 (31%) with apneas.

#### Diagnosis

**Fig. 2:**
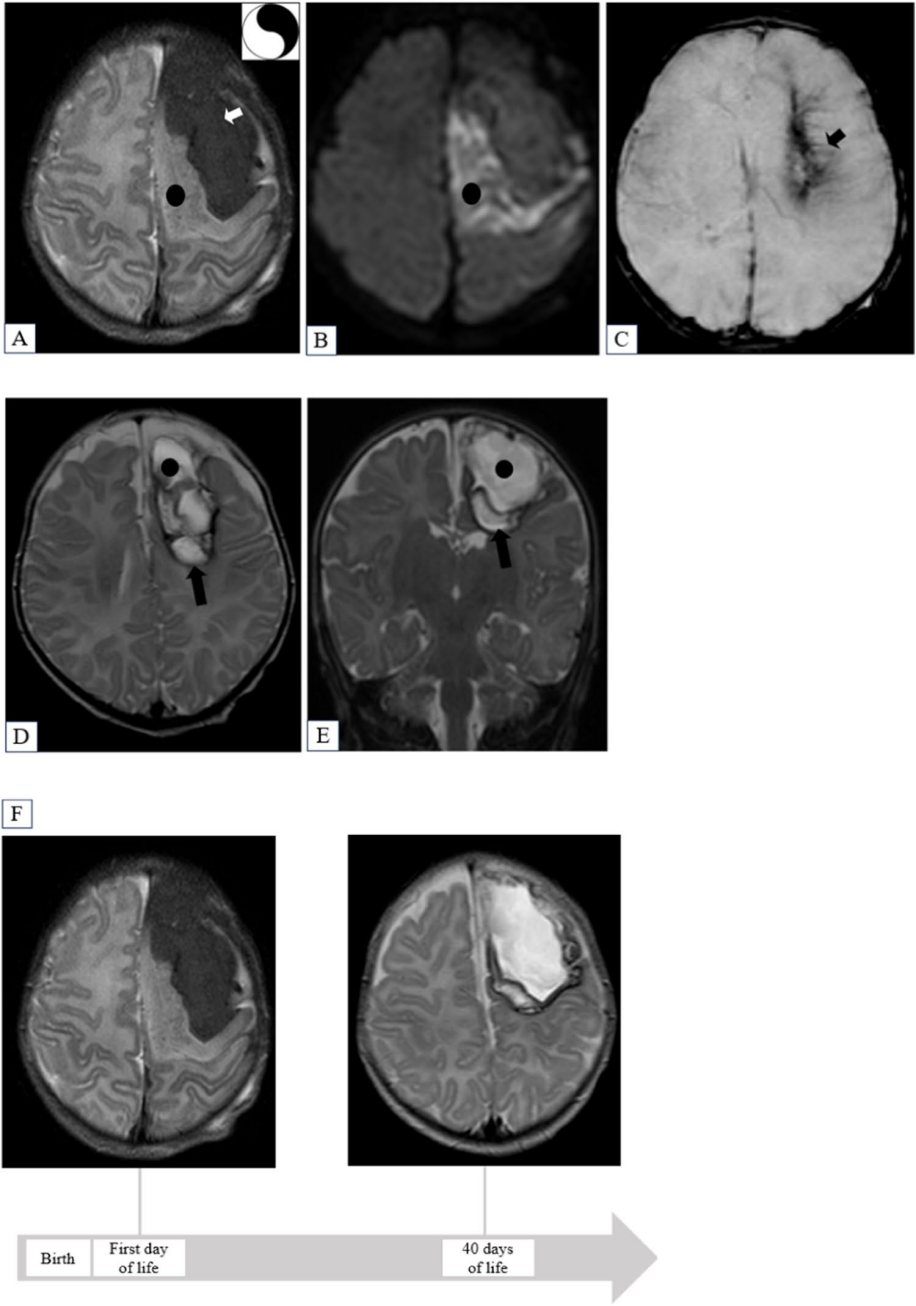
A term female newborn presented with focal motor clonic seizures at 14 h of life, which were refractory to phenobarbital but responded to phenytoin. MRI obtained on the first day of life (**A**, **B**, **C**) and after 40 days (**D**, **E**) and a timeline illustrating the evolution of the patient’s left frontal subpial hemorrhage as depicted on MRI T2-weighted imaging sequences (**F**) are shown. Her last clinical assessment at the age of 1 year and 9 months revealed a mild speech delay. MRI on 1st day: Axial T2WI (**A**), DWI (**B**), and T2*WI (**C**) display left frontal subpial hemorrhage (white arrow) and underlying cerebral infarction (black circle), resembling the yin-yang symbol; prominent medullary veins in the underlying white matter or “iris sign” (black arrow). Follow-up MRI after 40 days. Axial (**D**) and coronal (**E**) T2WI showing a peculiar pattern, with subpial cystic cavity (black circle) and underlying encephalomalacia (black arrow)

Most newborns (162/173, 94%) were diagnosed through brain MRI and/or cranial ultrasound, while 11 infants (6%) were identified with autopsy. Lesions were predominantly located in the temporal lobe (104/173, 60%). Concomitant infarction in parenchyma adjacent to SPH was observed in 145/162 (90%), and parenchymal hemorrhage was found in 102/162 (63%). Concomitant hemorrhage in other compartments was primarily intraventricular (42/158, 27%) and subdural (49/158, 31%). The Yin-Yang sign was reported in 47/162 infants (29%, Fig. 2A, B). Imaging biomarkers of medullary vein involvement, including the iris sign, were observed in 38/162 neonates (23%, Fig. 2C). Among 34 patients from cohort 3 with apneas, 24 (71%) presented a temporal lobe SPH (Table 3).

**Table 3:** SPH diagnosis with brain MRI findings at onset

		Cohort 1 *n* 10 *N* (%)	Cohort 2 *n* 163 *N* (%)	Subcohort 2 *n* 57 *N* (%)	Cohort 1 + 2 *n* 173 *N* (%)
Diagnosis	Brain MRI/cranial US	10/10 (100%)	152/163 (93%)	46/57 (81%)	162/173 (94%)
	Post-mortem	0	11/163 (7%)	11/57 (19%)	11/173 (6%)
SPH location	Frontal	3/10 (30%)	30/163 (18%)	9/57 (16%)	33/173(19%)
	Temporal	8/10 (80%)	96/163 (59%)	42/57 (74%)	104/173 (60%)
	Parietal	2/10 (20%)	33/163(20%)	7/57 (12%)	35/173 (20%)
	Occipital	3/10 (30%)	32/163 (19%)	8/57 (14%)	35/173 (20%)
Concomitant lesions/hemorrhages	Parenchymal cytotoxic Edema	10/10 (100%)	135/152 (89%)	46/46 (100%)	145/162 (90%)
	Parenchymal hemorrhage	10/10 (100%)	92/152 (60%)	29/45 (64%)	102/162 (63%)
	IVH	4/10 (40%)	38/148 (26%)	15/43 (35%)	42/158 (27%)
	Cerebellar hemorrhage/microbleeds	4/10 (40%)	14/148 (9%)	0	18/158 (11%)
	Epidural hemorrhage	0	2/148 (1%)	2/43 (5%)	2/158 (1%)
	Subdural hemorrhage	3/10 (30%)	46/148 (31%)	9/43 (21%)	49/158 (31%)
	Subarachnoid hemorrhage	2/10 (20%)	34/148 (23%)	1/43 (2%)	36/158 (23%)
Additional brain MRI findings	Multifocal SPH	1/10 (10%)	14/102 (14%)	4/38	15/112 (13%)
	Bilateral SPH	1/10 (10%)	6/51 (12%)	0	7/61 (11%)
	Mass effects	7/10 (70%)	53/82 (64%)	3/18 (17%)	60/92 (65%)
	Yin-Yang sign	10/10 (100%)	37/152 (24%)	1/46 (2%)	47/162 (29%)
	Iris sign	7/10 (70%)	31/152 (20%)	12/46 (26%)	38/162 (23%)
Flow sensitive brain MRI sequences	MRA	7/10 (70%)	48/93 (52%)	17/34 (50%)	55/103 (53%)
	MRV	3/10 (30%)	37/93 (40%)	18/34 (53%)	40/103 (39%)

*IVH* intraventricular hemorrhage, *MRA* magnetic resonance angiography, *MRI* magnetic resonance image, *MRV* magnetic resonance venography, *SPH* subpial hemorrhage, *US* ultrasound

#### Treatment

None of the patients received antithrombotic drugs. Treatment was conservative: at least one antiseizure medication was administered in 24/36 (66%) patients while 15/171 (9%) received a decompressive surgery.

#### Follow-up

Clinical follow-up data were available for 129/173 (75%) cases of the SP. Among these, 20/129 (16%) died: 1 patient for massive parenchymal hemorrhages, 7 for undefined comorbidities, and 12 for unspecified reasons.

At the last follow-up (median age 24 months, 3–168), 58/109 (53%) patients presented with a neurological impairment and 9/109 (8%) with post-stroke epilepsy or remote seizures.

In cohort 3, follow-up was available for 45 patients (9 from cohort 1 and 36 from cohort 2), 14 of whom died (31%). Among the remaining 31 patients, 16 (52%) exhibited neurological impairment, 3 (10%) developed post-stroke epilepsy (one of them also having neurological impairment), and 13 (42%) normal neurological findings.

Imaging follow-up was available for 82/173 (47%) patients, revealing subpial cysts in 22/82 (27%) and signs of encephalomalacia or tissue loss in 42/82 (51%) (Table 4) (Fig. 2D, E).

**Table 4:** Clinical and neuroradiological features at last follow-up

		Cohort 1 *n* 10 *N* (%)	Cohort 2 *n* 163 *N* (%)	Subcohort 2 *n* 57 *N* (%)	Cohort 1 + 2 *n* 173 *N* (%)
Clinical follow up	FU (in months), mean, median, range	17.62; 13; 3–36	37.9; 24; 4–168	39.07; 24;4–168	33.64; 24; 3–168
	Lost at FU	1/10 (10%)	43/163 (26%)	21/57 (37%)	44/173 (25%)
	Death	1/9 (1%)	19/120 (16%)	13/36 (33%)	20/129 (16%)
	Neurological impairment	7/8 (88%)	51/101 (50%)	9/23 (39%)	58/109 (53%)
	Epilepsy/remote seizure	1/8 (13%)	8/101 (8%)	2/23 (9%)	9/109 (8%)
Imaging follow-up	FU (in months), mean, median, range	2,52; 2; 0,2–7	NA	NA	NA
	Available data on imaging FU	8/10 (80%)	74/163 (43%)	8/57 (14%)	82/173 (47%)
	Subpial cyst	4/8	18/74 (24%)	1/8	22/82 (27%)
	Encephalomalacia/tissue loss/porencephaly	8/8	34/74 (46%)	4/8	42/82 (51%)

*FU* follow-up

### Factors associated with adverse outcome in the cohort 3 (n = 67)

LBW (*r* = 0.37, *p* value = 0.012) and CHD (*r* = 0.38, *p* value = 0.008) were associated with death, though the latter, based on a single patient, should be interpreted with caution. The occurrence of seizures (*r* = 0.28, *p* value = 0.043), neonatal infection/sepsis (*r* = 0.32, *p* value = 0.041), and parenchymal hemorrhage (*r* = 0.48, *p* value = 0.002) were associated with neurological impairment (Table 5).

**Table 5:** Within the subset of patients with available IPD (cohort 1 + subcohort 2, *n* = 67) correlation matrix with the Pearson’s correlation coefficient (*r*), the associated *p* value, and the total number of cases for each comparison are shown

	Death (*n* 14/67 pts)	Neurological impairment (*n* 17/67 pts)	Death or neurological impairment (*n* 31/67 pts)
Birth weight < 2500 g	***N* = 5**	*N* = 4	***N* = 9**
Seizures	***N* = 1**	***N* = 11**	*N* = 12
Neonatal infection or sepsis	*N* = 0	***N* = 4**	*N* = 4
Congenital heart disease	***N* = 1**	*N* = 0	*N* = 1
Parenchymal hemorrhage	*N* = 2	***N* = 8**	*N* = 10

*g* grams, *pts* patients

(Significant cases are highlighted in bold)

Additional significant positive associations include the temporal location of SPH with vaginal delivery (*r* = 0.33, *p* value = 0.048) and CHD with multifocal SPH (*r* = 0.39, *p* value = 0.02).

### Comparison between cohort 1 (n = 10) and subcohort 2 (n = 57)

As shown in Table 6, the following variables were more frequent in cohort 1: female gender, multifocal SPH, parenchymal hemorrhage, neurological impairment, and death or neurological impairment with Pearson’s *r* ranging from 0.31 to 0.63 and *p* values ranging from < 0.001 to 0.047.

**Table 6:** Comparison between cohort 1 and subcohort 2: total number of cases, the Pearson’s correlation coefficient (*r*), and the associated *p* value

	Cohort 1 (*n* = 10)	Subcohort 2 (*n* = 57)	Pearson Index *r* *	*p* value
Female gender	7/10	9/27	0.33	0.047
Multifocal SPH	6/10	7/56	0.38	0.009
Parenchymal hemorrhage	10/10	9/56	0.63	< 0.001
Neurological impairment	7/10	10/56	0.50	< 0.001
Death or neurological impairment	8/10	23/56	0.31	0.015

*pts* patients

*Please note that positive Pearson’s correlation indicates higher % of cases found in cohort 1 vs. cohort 2 with available IPD

## Discussion

To our knowledge, this review represents the first systematic analysis of risk factors, clinical and neuroradiological features, and outcomes of neonatal SPH. Additionally, clinical and neuroradiological factors associated with adverse outcomes were also illustrated.

The true incidence of SPH is unclear due to its historical inclusion with subarachnoid hemorrhage under the term “leptomeningeal hemorrhage” [17]. SPH predominantly affects term or late preterm newborns (92%).

A male predominance was observed, consistent with data from other neonatal stroke types, such as neonatal hemorrhagic stroke (NHS) and neonatal arterial ischemic stroke (NAIS) [18–20]. While previous studies suggested elevated endogenous testosterone may increase cerebral thromboembolism risk, its relevance in SPH remains to be determined [20].

Most newborns in our study had an uncomplicated birth, with a good Apgar score and birth weight > 2500 g.

Vaginal delivery was common in SPH (nearly 75%) compared to previous studies on NAIS and NHS, where cesarean sections, particularly emergency ones, were significant risk factors as a consequence of fetal compromise [20].

Birth trauma was frequent (26%), suggesting that vaginal delivery, particularly when assisted, may be a traumatic cause of pial-glial disruption and subsequent SPH [1, 3, 5, 11]. However, Cain et al. challenged this association, proposing that fetal distress, rather than birth trauma, is a key cause of SPH [6].

Asphyxia occurred in 12% of neonates, with need for neonatal resuscitation in 26% of cases. These conditions are known risk factors for NHS, NAIS, and in the largest case series of SPH [1, 20]. Despite this, it remains unclear whether they directly contribute to pathogenesis of SPH or merely represent signs and symptoms of an ongoing SPH.

Coagulopathy was a proposed etiology in SPH as well as in some cohorts of NHS [6]. Acute and/or transient coagulopathy, including thrombotic and hemorrhagic abnormalities, were noted in nearly a third of patients, but it remains uncertain if they are a cause of SPH or a consequence of an underlying inflammatory process [21, 22].

No major maternal, neonatal, or peripartum predisposing factors were found, suggesting a multifactorial pathogenesis for SPH. Nevertheless, an important traumatic role of vaginal delivery and birth trauma is plausible [23]. In support of this, SPH has also been reported along with other injury patterns in cases of abusive head trauma in young infants [24]. We speculate that pre- and peripartum conditions might act synergistically on a genetic and anatomic predisposing substrate, leading to the rupture of small vessels in the subpial space. Emerging evidence points to genetic factors, such as mutations in COL4A1 and COL4A2 genes, as relevant to neonatal intracranial bleeding [25]. Thus, we suggest performing genetic testing (e.g., WES/WGS in trio) in neonatal SPH to refine its etiological diagnosis.

Clinically, over one-third of patients presented with apneic events, another third with seizures, and nearly one-fifth had encephalopathy. Compared to other NHS studies, the prevalence of seizures and encephalopathy was lower, while for apneas were similar [18, 26]. Apneas in term neonates, especially if not associated with bradycardia, might recognize an ictal origin (autonomic seizures). Autonomic seizures have been linked to intracranial hemorrhage and should be considered in the differential diagnosis of apneic events especially in term neonates [27]. In fact, some studies reported neonatal ictal apneas, isolated or associated with other seizure types, as key signs of intracranial temporal bleeding suggesting an influence of the temporal cortex and/or amygdala on brainstem breathing centers [13]. The predominance of temporal lobe injury (60% of SP) could explain the high frequency of apneas and seizures.

In most studies, an electroencephalogram correlate of apneas was not reported, not allowing differentiation between ictal and non-ictal origins; thus, it is possible that some of the apneas could have been manifestations of autonomic or sequential seizures.

From a neuroradiological point of view, our study highlighted three key findings: the prevalence of temporal lobe involvement, the supratentorial location of SPH, and the presence of isolated SPH without parenchymal infarction in some patients.

Previous studies provided various explanations for the prevalence of temporal location in SPH. Huang et al. and Matsubara N. et al. suggested that the pterion, a large and relatively unprotected sutural confluence in neonates adjacent to the temporal lobe, is particularly vulnerable to vascular injury due to the shifting positions of cranial bones during vaginal delivery [5, 28]. We found a substantial and relevant correlation (*r* = 0.33, *p* = 0.048) between vaginal delivery and temporal SPH in the SP, potentially corroborating the role of vaginal delivery as a risk factor for temporal lobe SPH.

SPH was predominantly supratentorial, with infratentorial occurrences being rare. This might be due to different venous drainage patterns in infratentorial versus supratentorial regions, a hypothesis that requires further investigation.

Ten percent of all neonates had isolated SPH without adjacent parenchymal infarction. This finding seems to support the pathogenic hypothesis that the rupture of subpial vessels is the initial event in SPH. As in acute compartment syndrome, blood initially accumulates in the subpial space, leading in most, but not all, cases to compression/obstruction of cortical venous outflow and subsequent venous infarction of the underlying brain, possibly complicated by parenchymal hemorrhage.

SPH can result in significant long-term sequelae: over half of the neonates had neurological impairment and 8% developed post-stroke epilepsy.

We examined whether certain clinical and neuroimaging features were associated with death or neurological impairment (Table 5). Our analysis revealed a positive association between death and LBW and CHD. However, the association with CHD was derived from data involving a single patient and thus could not be considered statistically significant. The majority of deaths were attributable to severe comorbidities, making it challenging to ascertain the specific contribution of SPH. Consequently, further research is warranted to elucidate the mortality associated with SPH. Three risk factors were found to have a statistically significant positive correlation with neurological impairment: the occurrence of seizures, concomitant neonatal infection or sepsis, and the presence of parenchymal hemorrhage.

It is well established that neonatal seizures can adversely affect long-term neurodevelopmental outcomes, with severity also influenced by seizure burden, defined as the total duration of electrographic seizures over a given period [29]. In patients with HIE, longer seizure burdens have been associated with poorer motor and cognitive outcomes [30]. In this SP, data concerning the seizure burden were not reported. A better seizure characterization (including seizure burden and type) in future studies will help determine the role of seizures in worsening SPH neurodevelopmental outcomes.

Neonatal infection and sepsis are risk factors also associated with other types of neonatal stroke [31]. We hypothesize that their role in worsening long-term outcomes may be due to systemic involvement and inflammation.

Parenchymal hemorrhage showed the strongest correlation with neurological impairment. Consistent with this association, Zhuang et al. reported a worse outcome in SPH complicated by parenchymal hemorrhage [1]. Blood components can have multiple deleterious effects on ischemic brain parenchyma by enhancing inflammatory processes, altering tissue cytoarchitecture [32, 33], and consequently impairing long-term neurodevelopmental outcomes.

When comparing cohort 1 with subcohort 2, several significant differences emerged, with the limitations imposed by the small cohort 1 patients’ number (Table 6). In cohort 1, the incidence of parenchymal hemorrhage and multifocal SPH was notably higher, and we observed significantly worse outcomes.

These differences are likely multifactorial. However, the higher frequency of severe outcomes in our center cohort could be attributed to the increased prevalence of multifocal SPH and parenchymal hemorrhages, reinforcing the association observed in the entire SP. Our institution serves as a regional referral NICU, which may explain the higher prevalence of more severe forms of SPH and adverse outcomes in our cohort.

Limitations to this study include the heterogeneity of the data collected from selected articles. Data collection was constrained by their availability and, in most cases, it was not possible to ascertain the IPD. Only one author of the literature cohort answered our request to gather individual data. As a consequence, some findings are based on a limited number of children with SPH. As Sackett et al. have noted, a dropout rate exceeding 20% can present significant threats to validity [34]. When available, long-term outcomes may have been influenced by the presence of underlying ischemic or hemorrhagic infarction, making it difficult to determine the sole contribution of SPH. Moreover, due to its retrospective design, follow-up was not standardized, and a control group was lacking.

We acknowledge these limitations but believe that this work provides a valuable initial overview of the existing data on this rare condition in the literature. Further studies are needed to validate these findings.

## Conclusion

To our knowledge, this is the first systematic literature review on SPH. The understanding and characterization of SPH are expanding, and thus far, no major maternal, fetal, or neonatal risk factors have been identified. This suggests a multifactorial pathogenesis, with birth trauma associated with vaginal delivery likely playing a significant role.

We have identified a distinctive clinical presentation involving apnea (potentially of ictal origin) and seizures, along with a high prevalence of temporal lobe lesions. Prognostically, there is a notable prevalence of neurological impairment (53%). The occurrence of a parenchymal hemorrhage adjacent to SPH is strongly associated with adverse outcomes. Future studies are needed to further characterize SPH, assess long-term clinical outcomes, and determine and strengthen clinical and radiographic prognostic factors.

## Data Availability

The research data obtained from the included articles are available and can be requested from the corresponding author and will be made available when requested.

## Abbreviations

CHD: Congenital heart disease
FU: Follow-up
G: Grams
GW: Gestational weeks
HELLP: Hemolysis, elevated liver enzymes and low platelets
HIE: Hypoxic ischemic encephalopathy.
IPD: Individual patient data
IVH: Intraventricular hemorrhage
LBW: Low birth weight
MRA: Magnetic resonance angiography
MRI: Magnetic resonance imaging
MRV: Magnetic resonance venography
NAIS: Neonatal arterial ischemic stroke
NHS: Neonatal hemorrhagic stroke
Pts: Patients
PROM: Premature rupture of membranes
SEM: Structural equation model
SP: Study population
SPH: Subpial hemorrhage
US: Ultrasound

## Declarations

### Disclaimer

The funders had no role in the design of the study, in the collection, analyses, or interpretation of data, in the writing of the manuscript, and in the decision to publish the results.
